# Supplementary figures and images for: Fatty acid binding protein 5 regulates docetaxel sensitivity in taxane-resistant prostate cancer cells
Source: PLoS One. 2023 Oct 5;18(10):e0292483. doi: 10.1371/journal.pone.0292483 (PMC10553314; doi:10.1371/journal.pone.0292483)

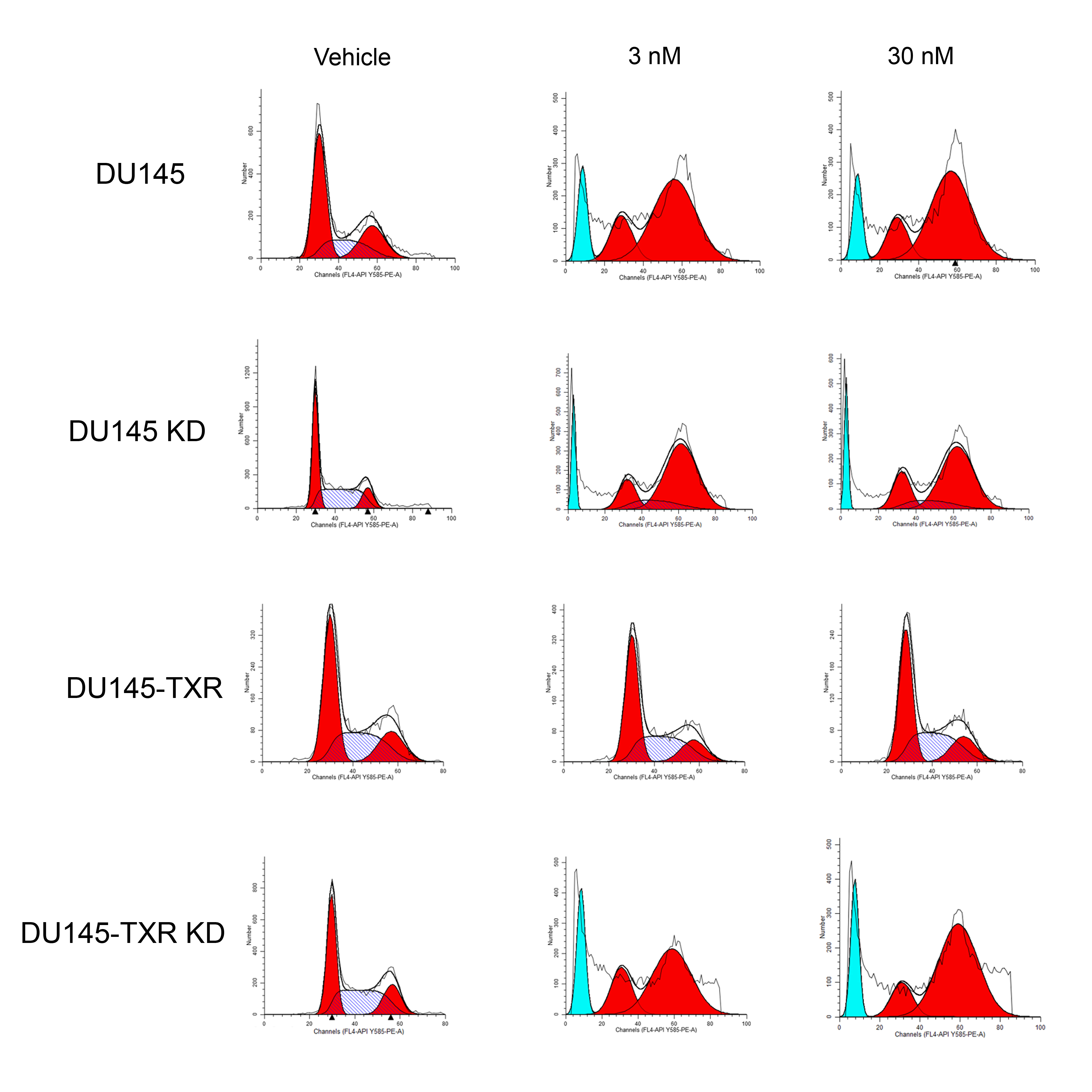

Supplement: S1 Fig — G0/G1, G2/M, and S phase populations were resolved on a linear scale using ModFit LX software. G0/G1 and G2/M correspond to a PI signal of 30 and 60%, respectively. S-phase is represented by the shaded segments while apoptotic cells are contained within the solid teal curves and assigned a PI signal of 10%. (TIF) [file pone.0292483.s001.tif]

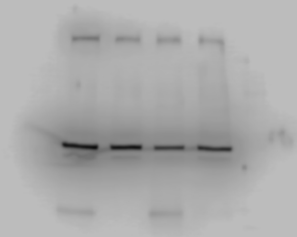

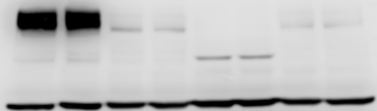

Supplement: S1 Raw images — (PDF) [file pone.0292483.s002.pdf]
